# fMRI changes during multi-limb movements in Parkinson’s disease

**DOI:** 10.3389/fnhum.2023.1248636

**Published:** 2023-11-09

**Authors:** Jae Woo Chung, Abigail E. Bower, Ibrahim Malik, Justin P. Martello, Christopher A. Knight, John J. Jeka, Roxana G. Burciu

**Affiliations:** ^1^Department of Kinesiology and Applied Physiology, University of Delaware, Newark, DE, United States; ^2^Center for Biomedical and Brain Imaging, University of Delaware, Newark, DE, United States; ^3^Department of Neurosciences, Christiana Care Health System, Newark, DE, United States; ^4^Interdisciplinary Neuroscience Graduate Program, University of Delaware, Newark, DE, United States

**Keywords:** Parkinson’s disease, fMRI, force, coordination, multi-limb

## Abstract

**Background:**

While motor coordination problems are frequently reported among individuals with Parkinson’s disease (PD), the effects of the disease on the performance of multi-limb movements and the brain changes underlying impaired coordination are not well-documented.

**Objective:**

Functional magnetic resonance imaging (fMRI) was used to examine differences in brain activity during a task that involved the coordination of non-homologous limbs (i.e., ipsilateral hand and foot) in individuals with and without PD.

**Methods:**

Participants included 20 PD and 20 healthy control participants (HC). They were instructed to generate force in a coordinated manner by simultaneously contracting their ipsilateral hand and foot. PD were tested off their antiparkinsonian medication and on their more affected side, whereas the side in controls was randomized.

**Results:**

Although both groups were able to coordinate the two limbs to produce the expected level of force, PD had a slower rate of force production and relaxation compared to HC. Additionally, their globus pallidus and primary motor cortex were underactive, whereas their pre-supplementary motor area (pre-SMA) and lateral cerebellum were overactive relative to HC. Importantly, in PD, the fMRI activity within the pre-SMA correlated with the rate of force decrease.

**Conclusion:**

Multi-limb force control deficits in PD appear to be related to widespread underactivation within the basal ganglia-cortical loop. An overactivation of higher-level motor regions within the prefrontal cortex and lateral cerebellum may reflect increased cognitive control and performance monitoring that emerges during more complex motor tasks such as those that involve the coordination of multiple limbs.

## 1. Introduction

Parkinson’s disease (PD) is a progressive neurodegenerative disorder characterized by the gradual loss of dopamine-producing neurons (Fearnley and Lees, 1991; Obeso et al., 2008; Kordower et al., 2013). While the disease is primarily recognized for its four cardinal signs (i.e., bradykinesia, rest tremor, rigidity, and postural instability) (Jankovic, 2008; Postuma et al., 2015; Obeso et al., 2017; Armstrong and Okun, 2020), it is essential to acknowledge another significant motor manifestation that profoundly affects daily activities in PD: impaired interlimb coordination (Lazarus and Stelmach, 1992; Verschueren et al., 1997; Verheul and Geuze, 2004; Roemmich et al., 2013; Kellaher et al., 2022). In the field of movement sciences, interlimb coordination refers to the harmonious control of movements across different limbs to achieve a desired motor goal (Carpenter, 1968; Shirota et al., 2016). It can involve simultaneous or sequential movements in tasks that utilize either two homologous limbs (e.g., hands for utensil use or tying shoelaces, legs during walking) or two non-homologous limbs (e.g., arms and legs during walking) (Shirota et al., 2016).

Some of the brain regions that work together in a coordinated manner to facilitate multi-limb tasks in healthy individuals are the basal ganglia, primary sensorimotor cortex (M1S1), supplementary motor area (SMA), premotor cortex, cingulate motor cortex, and cerebellum (Debaere et al., 2001, 2004; Swinnen, 2002). In PD, single-limb movements (particularly upper limb movements) have been extensively studied to gain insight into the effects of dopaminergic dysfunction on the neural control of movement (Herz et al., 2014, 2021). The neural correlates of multi-limb motor impairment in PD, however, are not as well-understood. Previous brain imaging studies of single-limb movements demonstrated changes in the activation of several brain structures across the basal ganglia- and cerebellar-cortical motor loops in PD compared to healthy aging (Herz et al., 2014, 2021). A recent quantitative meta-analysis of functional neuroimaging studies testing primarily isolated movements of the upper extremity found a mixed pattern of underactivation and overactivation in these areas (Herz et al., 2021). Specifically, PD were found to have reduced motor-related activity within the posterior putamen, cerebellum, primary motor cortex (M1), and SMA, and increased motor-related activity in the cortical regions located directly anterior to M1 and SMA (Herz et al., 2021). Functional magnetic resonance imaging (fMRI) studies examining the neural correlates of coordinated movements in PD are very scarce. In a study examining the neural correlates of in-phase and anti-phase bimanual movements, PD demonstrated more difficulties performing coordinated movements when the movements were anti-phase and that this was associated with reduced activity within the basal ganglia and SMA, and increased activity within the premotor cortex, inferior frontal gyrus, precuneus, M1 and cerebellum (Wu et al., 2010). Collectively, previous imaging studies provide valuable insights into the neural correlates of motor symptoms in PD but also underscore the need for further investigation into the realm of multi-limb movements. By further studying multi-limb movements, the field can gain a more comprehensive understanding of how these interconnected brain networks contribute to the coordination of movements and how their dysfunction leads to deficits in PD. It is important to recognize that numerous everyday activities require the coordinated recruitment of multiple limbs, emphasizing the practical significance of investigating the brain regions orchestrating these coordinated actions.

Considering this important gap in the knowledge, the aim of our study was to use fMRI to investigate, for the first time, PD-related changes in brain activity during a multi-limb task that involved rapid and simultaneous contractions of the hand and foot on the ipsilateral side. The choice to study the movements of the ipsilateral hand and foot was motivated by the fact that interlimb coordination on non-homologous movements is important for a range of everyday behaviors including locomotion (Donker et al., 2001; Balter and Zehr, 2007). PD is often associated with reduced or asymmetric arm swing (Siragy et al., 2020; Siragy and Nantel, 2020), and this reduction in arm swing tends to disrupt the natural coordination between the arms and legs that occurs during locomotion (Zampieri et al., 2010; Huang et al., 2012). Furthermore, PD symptoms are more pronounced on the same side of the body, including the hand and foot on that side (Toth et al., 2004; Cotogni et al., 2021).

To achieve the goal of our study, we employed a well-established force fMRI paradigm that has been validated in previous upper limb studies of PD (Prodoehl et al., 2010; Spraker et al., 2010; Burciu et al., 2015, 2016a,b; Neely et al., 2015; Planetta et al., 2015; Chung et al., 2018) and more recently applied to studying the brain changes in PD during ankle dorsiflexion (Chung et al., 2023a). Over time, this protocol highlighted two key characteristics of the blood-oxygen-level-dependent (BOLD) fMRI response during unilateral submaximal isometric force controls in PD. Firstly, this response tends to be diminished in PD in the basal ganglia, M1, and cerebellum during single-limb movements involving either the hand or the foot, as evidenced by several studies involving both *de novo* and medicated PD patients (Prodoehl et al., 2010; Spraker et al., 2010; Burciu et al., 2015, 2016a,b; Neely et al., 2015; Planetta et al., 2015). Secondly, during unimanual force production, this response exhibited a decline over the course of 1 year of disease progression in PD, while remaining unchanged by 1 year of healthy aging (Burciu et al., 2016a). These results indicate that changes in force control and the corresponding patterns of brain activity have the potential to serve as biomarkers for tracking disease progression in PD. Furthermore, the observed sensitivity of this paradigm in detecting disease-related effects in both cross-sectional and longitudinal studies provides additional support for its applicability in assessing multi-limb coordination in PD.

In this multi-limb force control study, we hypothesized a decrease in fMRI activity in PD compared to controls within the basal ganglia and M1, consistent with findings from single-limb movements. Additionally, we anticipated a further reduction in fMRI signal in PD within higher-order motor regions, specifically the premotor cortex and cerebellar hemispheres, which play crucial roles in movement planning and coordination. Finally, we hypothesized that individuals with PD would exhibit impaired force generation and force relaxation, similar to the findings observed in previous single-limb studies that tested isolated movements of the hand or foot. Additionally, we anticipated the presence of coordination deficits in PD compared to controls.

## 2. Methods

### 2.1. Participants and clinical assessments

The study included two groups: 20 individuals diagnosed with PD and a comparison group of 20 healthy older adults. Seventeen individuals from each group also took part in a prior imaging study aimed at investigating fMRI changes in PD during single-limb movements that required isometric ankle dorsiflexion (Chung et al., 2023a) (~ 75% overlap of participants between the two studies). The decision regarding the sample size was guided by prior fMRI studies on upper-limb force control in PD which often employed approximately 20 participants in each group (Neely et al., 2015; Planetta et al., 2015; Burciu et al., 2016a), and a power analysis based on fMRI data collected as part of the aforementioned single-limb, ankle dorsiflexion study (Chung et al., 2023a). The patient group consisted of 20 individuals diagnosed with PD by a movement disorder specialist according to established diagnostic criteria (i.e., United Kingdom PD Society Brain Bank Criteria). The patients were recruited from the ChristianaCare Neurology Specialists Clinic in Newark, Delaware, and the University of Delaware Participant Recruitment Registry for Parkinson’s disease Research. The exclusion criteria for patients were as follows: (1) a history of other neurological or psychiatric disorders, (2) an atypical form of parkinsonism, (3) dementia, (4) a history of cancer that required chemotherapy and/or radiotherapy, (5) deep brain stimulation surgery. T2-weighted MRI scans were also collected as part of this study for the purpose of excluding secondary causes of parkinsonism and/or other neurological events (e.g., vascular lesions, demyelinating lesions, tumors, etc.). The comparison group comprised of 20 healthy older adults without any history of neurological, psychiatric, or musculoskeletal disorders. HC were recruited via advertisements in the Newark area. Importantly, PD and HC were matched at the group level for age, sex, cognitive status, and tested side (Table 1). PD participants were tested on their more affected side, while the tested side in controls was randomized (more details in the force acquisition section). All participants were tested in the morning to minimize the effects of the time of the day and fatigue on task performance. PD patients were tested following an overnight withdrawal from antiparkinsonian medication (12–14 h after the last dose of PD medication). Information regarding the total Levodopa Equivalent Daily Dose (LEDD) for the patient group is available in Table 1. The Montreal Cognitive Assessment Test (MoCA) (Nasreddine et al., 2005) is a widespread cognitive tool that was used here to determine the cognitive status of each group. The motor section of the Movement Disorder Society Unified Parkinson’s Disease Rating Scale (MDS-UPDRS-III) (Goetz et al., 2008) was used to evaluate various aspects of PD and to rule out motor symptoms in control participants. The ratings for specific MDS-UPDRS-III hand and foot items were used to calculate sub-scores that describe the severity of motor symptoms in the tested hand/foot. Written informed consent was obtained from all participants. The study protocol was approved by the University of Delaware’s Institutional Review Board and conducted according to the Declaration of Helsinki.

**Table 1:** The table lists the clinical characteristics of PD and controls.

**Variable**	**HC**	**PD**	***p*-value**
N	20	20	-
Age (Y)	63.85 (± 9.46)	67.65 (± 7.93)	0.177
Sex (M | F)	10 M | 10 F	12 M | 8 F	0.525
Handedness (L | R)	3 L | 17 R	3 L | 17 R	1.000
Tested Side (L | R)	6 L | 14 R	11 L | 9 R	0.110
Tested Side (D | ND)	13 D | 7 ND	12 D | 8 ND	0.744
More Affected Side (L | R)	-	11 L | 9 R	-
Disease Duration (M)	-	56.70 (± 43.28)	-
Hoehn and Yahr Stage	-	2.00 (± 0.56)	-
Total LEDD	-	606.25 (± 573.94)	-
MDS-UPDRS-III, Total	1.90 (± 2.22)	30.90 (± 10.18)	<.001
MDS-UPDRS-III, Tested Hand	0.50 (± 0.95)	10.00 (± 4.32)	<.001
MDS-UPDRS-III, Tested Foot	0.15 (± 0.37)	4.15 (± 2.32)	<.001
MOCA	27.00 (± 2.85)	27.05 (± 2.41)	0.827

The last column lists the *p*-value corresponding to the statistical analyses described in the manuscript. Data represents the count or mean (± 1 standard deviation). PD patients were tested in the “off’ state, following an overnight withdrawal from PD medication. Disease duration is defined as time since diagnosis. D, dominant; F, females; HC, healthy controls; H & Y, Hoehn and Yahr Staging Scale; LEDD, levodopa equivalent daily dose; L, left; M, males; M, months; MDS-UPDRS-III, the motor section of the Movement Disorder Society Unified Parkinson’s Disease Rating Scale; MOCA, Montreal Cognitive Assessment; ND, non-dominant; PD, Parkinson’s disease; R, right; Y, years.

### 2.2. Force data acquisition and fMRI paradigm

**Figure 1:**
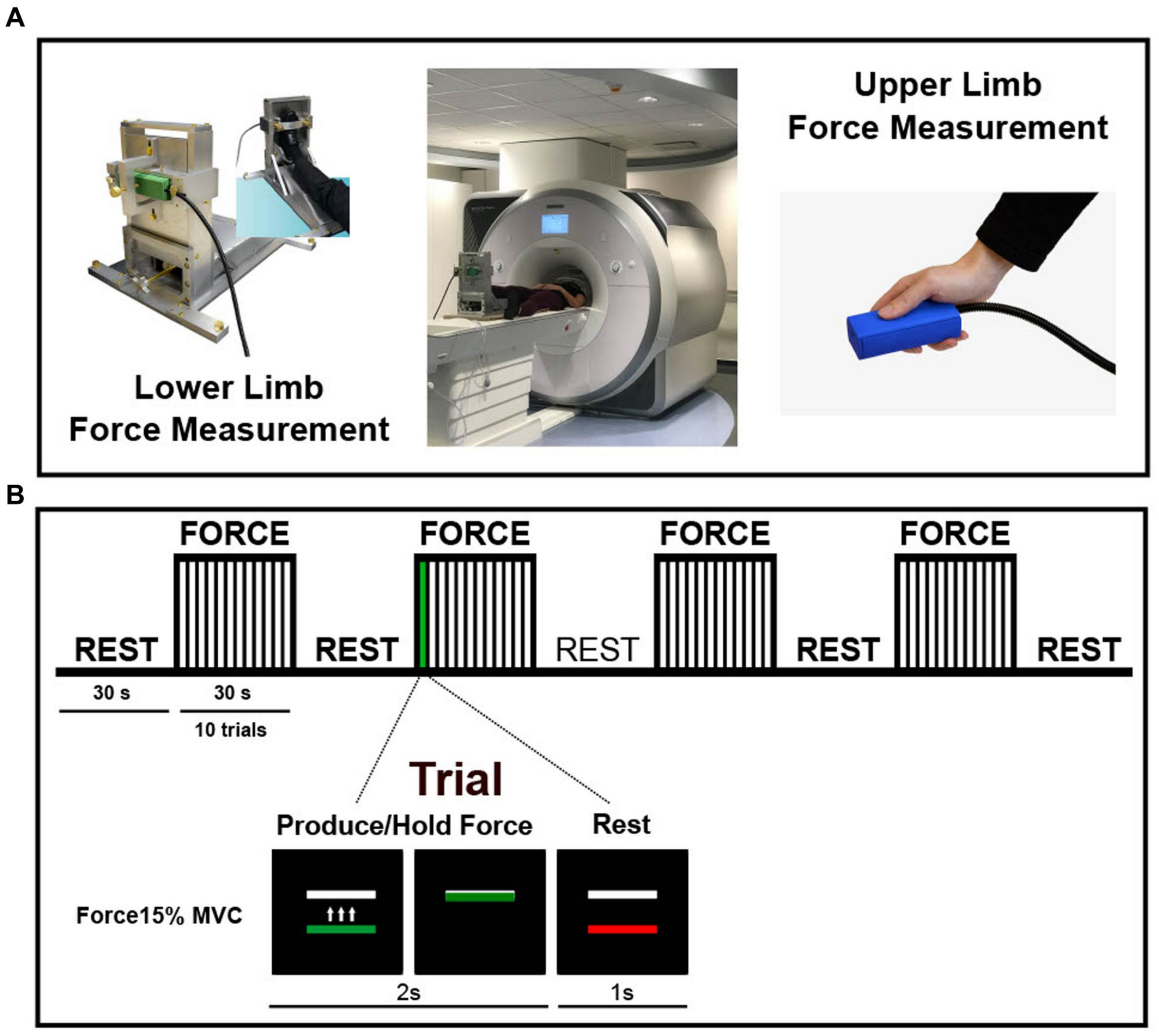
Experimental setup and paradigm. **(A)** An MRI-compatible foot device along with two force sensors for the hand and foot used for measuring the maximum voluntary contraction (MVC) and to produce force. **(B)** fMRI block paradigm. The multi-limb coordination task began with 30 s of rest, followed by 4 cycles consisting of 30 s force +30 s rest. Each of the four force blocks contained 10 trials. During a trial, participants had to coordinate the ipsilateral hand and foot (see methods for more information on how the tested side was selected) to produce force for 2 s and then relax for 1 s. A horizontal white bar remained stationary on the screen and represented the force target which was set for each participant to represent 15% of MVC. Force generation was cued by a change in the color of a force bar located below the target bar. When the force bar turned green, participants had to use both hand and foot to bring the force bar on top of a white bar. When the force bar turned red, participants relaxed their tested hand and foot. Visual feedback on task performance was provided. The moving force bar represented the sum of forces exerted by the hand and foot.

Force data inside the MRI scanner was generated using two custom MRI-compatible force transducers with a resolution of 0.025 N: one measuring the force produced by the hand, and one measuring the force produced by the foot (Neuroimaging Solutions, Gainesville, FL) (Figure 1A). Force signals from the two sensors were transmitted through a fiber-optic cable to a SI155 Micron Hyperion Optical Sensing Interrogator (Micron Optics, Atlanta, Georgia) which digitized the force data at 50 Hz. The data was collected with a custom data collection software written in LabVIEW (National Instruments, Austin, TX). Participants produced force with their tested hand by gripping the force sensor between their thumb and the other fingers. While pushing on the hand sensor with their thumb, they concurrently generated force with their foot by dorsiflexing their ankle. For measuring the isometric force during ankle dorsiflexion, we used a foot sensor that was placed at the back of a custom-made MRI-compatible foot device as seen in Figure 1A. The foot device has been used for the first time in a recent fMRI study of PD and healthy older adults performing single-limb movements (i.e., ankle dorsiflexion) and helped map the functional activity of the lower limb brain circuit in a reliable way while keeping the head motion minimal (Chung et al., 2023a). The tested foot was stabilized in the device with an adjustable strap placed over the metatarsals. The dorsiflexion of the ankle led a piston at the back of the foot device to apply a compressive force to the force sensor. Online visual feedback of the force output was projected onto a 32″ 1920 × 1,080 widescreen LCD display with a 120 Hz refresh rate located behind the MRI bore/participant’s head. The display was visible to participants via a mirror mounted on the head coil.

Participants completed an isometric force control task that required them to coordinate the ipsilateral hand and foot to produce force at 15% of their MVC. Prior to entering the MRI scanner, participants practiced the task. First, each participant performed three brief MVC trials. Each trial required participants to produce maximum force with both hand and foot simultaneously for 5 s. An average MVC of the three trials was calculated based on the summation of the hand and foot forces and was later used to normalize force demands across participants at 15% of their MVC. A representation of the fMRI paradigm is shown in Figure 1B. The fMRI block design proceeded as follows: 30 s of initial rest followed by four cycles of 30 s visually guided force task and 30 s of rest. Throughout the scan, participants viewed a black screen with two horizontal bars: a white bar positioned at the top that represented their force target (15% of their MVC) and a colored bar positioned at the bottom that acted as a cue. The bar turned green to cue the participant to produce force and it turned red to cue the participant to release the force and rest. In total, there were four force blocks of trials, with ten 3-s trials per block (1 trial = 2 s of force production +1 s of rest). Participants were instructed to produce force with both hand and foot simultaneously, to reach the target as quickly as possible, and to maintain a steady level of force while the bar was green. Importantly, the force displayed on the screen was the sum of the forces exerted by the hand and foot. PD patients were tested on their more affected side, whereas the tested side in controls was randomized so that there would be no statistically significant group differences in the ratio of people tested on their left/right side or dominant/non-dominant side.

### 2.3. Force data analysis

The analysis of the force data was performed using custom scripts in MATLAB R2021b (The Mathworks, Natick, MA). Briefly, data were filtered using a 6th-order Butterworth filter with a cutoff frequency of 15 Hz. Next, consistent with previous studies employing this force paradigm (Neely et al., 2013a; Planetta et al., 2015; Chung et al., 2023b), four points were defined for each trial: (1) onset of force, (2) onset of the steady period during which the colored bar matched the white target bar, (3) offset of the steady period, and (4) offset of force. Based on these four points, the following measures were calculated for each trial and then averages across all trials: normalized force amplitude (% MVC), the standard deviation of the force amplitude (% MVC), constant error (N), absolute error (N), rate of force increase (N/s), rate of force decrease (N/s), force amplitude calculated separately for the hand and foot (% total force amplitude), and time lag between the onset of force in the hand and foot (ms).

### 2.4. MRI data acquisition

All scans were collected on a 3 T Magnetom Prisma whole-body MRI scanner from Siemens equipped with a 64-channel head coil. fMRI scans were acquired using a single-shot gradient echo-planar imaging pulse sequence (TR = 2,500 ms, TE = 30 ms, 43 slices, flip angle = 80°, GRAPPA parallel imaging factor 2, FOV = 240 × 240 mm, resolution = 3 × 3 × 3 mm). High-resolution structural MRI scans were acquired using a T1-weighted sequence (TR = 2000 ms, TE = 2.99 ms, 208 slices, flip angle = 8°, GRAPPA parallel imaging factor 2, FOV = 256 × 256 mm, resolution = 0.8 × 0.8 × 0.8 mm). T2-weighted scans were also collected to rule out neurological diseases other than PD (TR = 2,500 ms, TE = 371 ms, 208 slices, flip angle = variable, GRAPPA parallel imaging factor 3, FOV = 256 × 256 mm, resolution = 0.8 × 0.8 × 0.8 mm).

### 2.5. fMRI data analysis

Consistent with previous fMRI research testing isometric force control during unilateral movements of the hand/foot only, PD (Prodoehl et al., 2010; Spraker et al., 2010; Burciu et al., 2015, 2016a,b; Neely et al., 2015; Planetta et al., 2015; Chung et al., 2018, 2023a), the fMRI and T1-weighted scans of participants who were tested on their left side were flipped in the left–right plane prior to processing. The fMRI data were analyzed using Unix shell scripts and the following MRI software packages and toolboxes: AFNI (Analysis of Functional Neuro Images),^1^ SPM12 (Statistical Parametric Mapping,^2^ SUIT (Spatially Unbiased Infratentorial Template).^3^ The fMRI analysis consisted of the following standard preprocessing steps: (a) skull stripping of the structural scan, (b) despiking of the fMRI scan, (c) slice-timing correction of the fMRI scan, (d) 3D rigid-body motion correction of the fMRI scan, (e) motion scrubbing by removing TR-to-TR motion > 0.5 mm, (f) coregistration of the fMRI and structural scans, (g) division of the fMRI signal in each voxel at each point in the time series by the mean signal in that voxel across the scan, (h) spatial normalization of the scans to the 152MNI template for a standard whole-brain analysis and to the SUIT template for a cerebellum- and brainstem-optimized analysis (Diedrichsen, 2006), (i) smoothing of the spatially normalized fMRI scans with a 4-mm Full Width Half Max (FWHM) Gaussian kernel. Following preprocessing, the fMRI signal during the four 30-s force blocks was modeled using a boxcar regressor convolved with the canonical hemodynamic response function. The six head motion parameters estimated during the motion correction step were included in the statistical analysis as regressors of no interest. Finally, we performed a voxel-wise Independent *T*-Test analysis to compare multi-limb coordination-related activity between PD and HC. The analysis was corrected for Type I error with a Monte Carlo simulation using 3dClustSim in AFNI. Active regions had to meet a threshold of *p* < 0.005 and a cluster size of 54 μL, corresponding to a *p* < 0.05, corrected for multiple comparisons using the family-wise error (FWE) correction. Brain regions where fMRI activity differed between groups were labeled using the basal ganglia human area template (BGHAT), the human motor area template (HMAT), the probabilistic atlas of the human cerebellum (SUIT), and the automated anatomical labeling atlas (AAL) (Tzourio-Mazoyer et al., 2002; Diedrichsen, 2006; Mayka et al., 2006; Prodoehl et al., 2008; Diedrichsen et al., 2009).

### 2.6. Statistical analysis

The statistical analysis of the clinical and force data was performed in SPSS 28.0 (IBM, New York). First, all outcome measures were assessed for normality and equal variance with Shapiro–Wilk and Levene’s Tests. The results of these tests prompted the choice of parametric or non-parametric statistical testing. Categorical data (see Table 1) were compared between groups using a Chi-Square test. All continuous measures except for age were compared between groups using the Mann–Whitney *U* Test. Age differences were compared between groups using an Independent *T*-Test. Finally, percent signal change (PSC) was calculated from key motor regions where the fMRI activity differed between groups and correlated in PD with the force measures that showed group differences. The calculation of PSC was based on the 15-s period spanning 6 TRs at the end of each force block based on previous force control studies in PD (Spraker et al., 2010; Burciu et al., 2016a; Chung et al., 2018, 2023a). The relationship between PSC and force performance was assessed using partial correlations controlling for MVC. Results were significant if *p*-values < 0.05.

## 3. Results

### 3.1. Clinical and force measures

In the present study, several sociodemographic and clinical measures were examined to assess potential group differences. Table 1 summarizes these measures for each group and the between-group statistics. There were no significant differences between groups in age, sex, cognitive status, handedness, and tested side based on body side or dominance (*p*-values > 0.05; Table 1). As expected, group differences were found in the total MDS-UPDRS-III score and the sub-scores of MDS-UPDRS-III for the tested hand and foot, respectively, (*p*-values < 0.001; Table 1). Force measures are summarized in Table 2. There were no differences between groups in the MVC which in this study represented the maximum force produced simultaneously by the ipsilateral hand and foot (*p* = 0.455). Other measures that did not differ significantly between groups are the normalized force amplitude during the steady period, SD of the normalized force amplitude, constant error, absolute error, the contribution of the hand and foot to the total force amplitude, and the time lag between the onset of force in each limb (*p*-values > 0.05; Table 2). These results indicate that both groups were able to coordinate the two limbs to produce the required force level and that the force variability, movement error, and hand-foot contribution to the total force were similar between groups. While PD were not necessarily more variable and less accurate than HC, they were slower. PD had a reduced rate of force increase (*p* = 0.027) and rate of force decrease relative to HC (*p* = 0.007) (Table 2).

**Table 2:** The table lists the results of the force analysis.

Variable	HC	PD	*p*-value
MVC (N)	133.20 (±66.01)	118.20 (±29.60)	0.455
Normalized force amplitude (% MVC)	14.99 (±1.51)	14.48 (±2.00)	0.417
SD of normalized force amplitude (% MVC)	0.90 (±0.39)	0.89 (±0.56)	0.402
Constant error (N)	−0.06 (±1.73)	−1.15 (±2.34)	0.402
Absolute error (N)	1.56 (±1.13)	1.61 (±2.12)	0.224
Rate of force increase (N/s)	48.90 (±31.48)	31.91 (±16.60)	0.027
Rate of force decrease (N/s)	−74.50 (±31.54)	−47.71 (±26.28)	0.007
Foot force amplitude (% Total force amplitude)	57.96 (±15.97)	67.40 (±15.50)	0.066
Hand force amplitude (% Total force amplitude)	42.04 (±15.97)	32.60 (±15.52)	0.066
Time lag (Hand Onset – Foot Onset) (ms)	−75.85 (±92.51)	−51.98 (±151.36)	0.551

The numbers listed in the table represent the mean ± 1 standard deviation. PD patients were tested in the “off’ state, following an overnight withdrawal from PD medication. HC, healthy controls; MVC, Maximum voluntary contraction; N, Newtons; PD, Parkinson’s disease.

### 3.2. fMRI results

**Figure 2:**
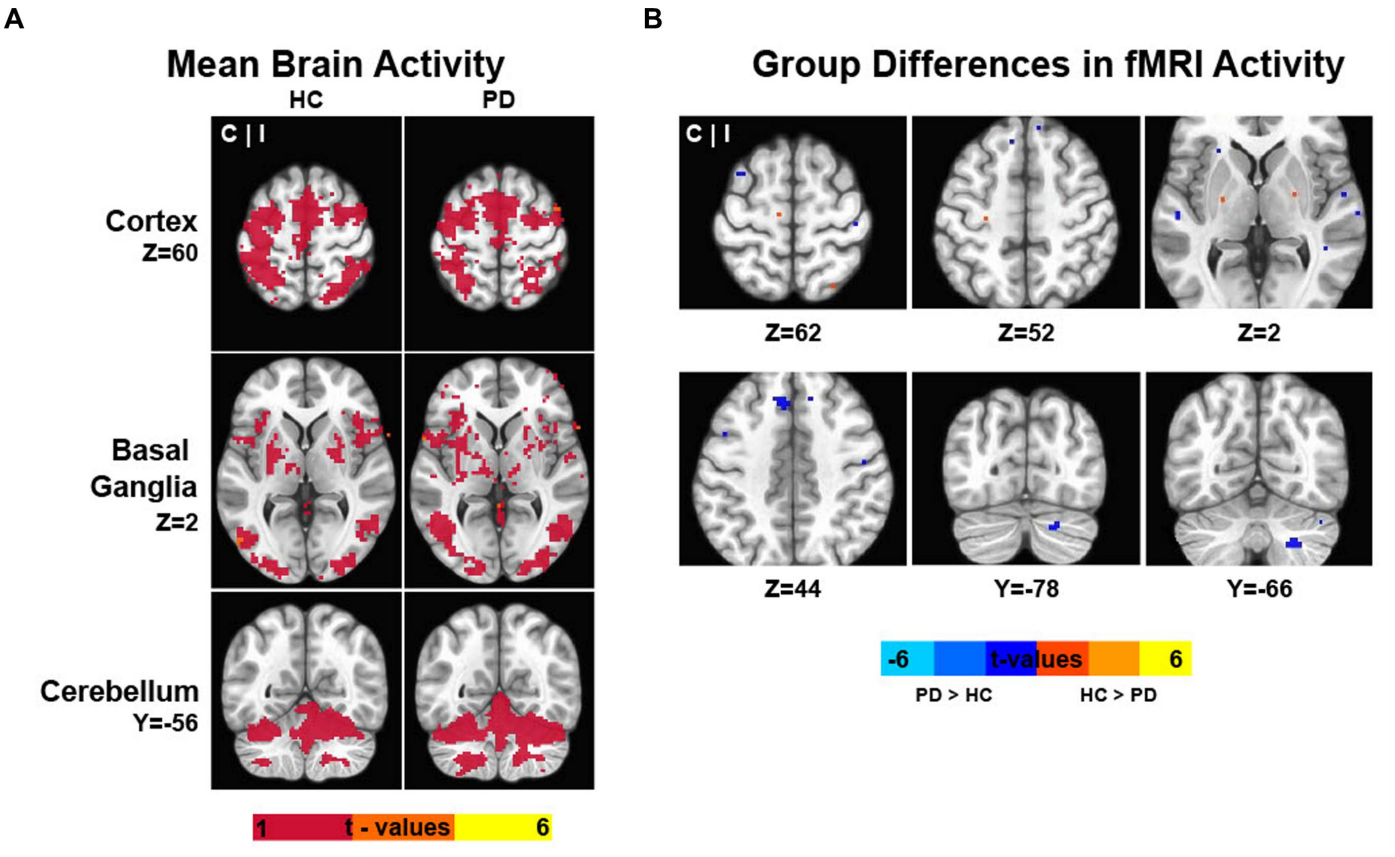
The figure illustrates the results of the whole-brain and cerebellum/brainstem-optimized analyses. **(A)** Data represent the mean fMRI activity during the multi-limb coordination task for PD and control participants. **(B)** Group differences in fMRI activity. Warm colors indicate the brain regions where healthy controls (HC) have greater force-related fMRI activity during the coordination task than PD. By contrast, cold colors indicate the brain regions where PD patients have greater force-related fMRI activity during the coordination task than HC. The fMRI results are overlaid on the MNI template. The color bars indicate the intensity of the fMRI results in T-values. C, contralateral to the side producing force; HC, healthy controls; I, ipsilateral to the side producing force; PD, Parkinson’s disease.

Figure 2A shows the activation maps during the multi-limb task for HC and PD. In both groups, producing force by contracting the ipsilateral hand and foot simultaneously resulted in an increase in BOLD fMRI signal in multiple premotor and sensorimotor cortical structures, the nuclei of the basal ganglia, thalamus, and several lobules within the anterior and posterior lobes of the cerebellum. As expected, the fMRI activity was predominantly contralateral in the cortex and ipsilateral in the cerebellum. Between-group results from the whole-brain voxel-wise fMRI analysis are depicted in Figure 2B. Overall, PD exhibited a mixed pattern of both hypoactivation and hyperactivation. Their performance on the multi-limb coordination task was associated with reduced fMRI activity compared to HC in the globus pallidus external segment bilaterally, and the contralateral hand and foot area of M1, respectively, (Figure 2B and Table 3). Compared to HC, PD had increased fMRI activity of cortical premotor regions including the contralateral pre-supplementary motor area (pre-SMA), prefrontal regions such as the superior frontal gyrus bilaterally, and several ipsilateral brain regions located in the lateral cerebellum (lobules crus I-II, VIIb, VIIIa, and dentate nucleus) (Figure 2B and Table 3). In addition to a more active prefrontal cortex and cerebellum, PD also had more active temporal cortices (see Table 3).

**Table 3:** The table describes the MRI results in terms of anatomical location, side of activation, cluster size, and intensity (*t*-value).

Anatomical regions	Side	Peak X Y Z MNI coordinates			Cluster size	*T*-value
		X	Y	Z		
HC > PD						
Inferior temporal gyrus	C	−45	−33	−21	8	3.14
GPe	C	−24	−9	3	4	3.18
GPe	I	24	−6	3	2	3.15
Middle occipital gyrus	I	42	−75	21	4	3.04
Superior temporal gyrus	I	57	−30	21	4	3.35
M1 hand area	C	−27	−21	54	4	3.36
M1 foot area	C	−9	−18	63	4	3.24
PD > HC						
Pre-SMA, superior frontal gyrus	C	−6	27	45	82	−5.24
Insula	C	−39	6	−6	21	−3.06
Insula	C	−30	12	−9	19	−3.51
Para hippocampal gyrus, fusiform gyrus	I	27	−6	−33	10	−3.39
Middle temporal gyrus	C	−57	−6	−21	14	−3.33
Superior temporal gyrus	C	−54	−18	3	7	−3.11
Inferior frontal gyrus, insula	C	−42	18	9	7	−3.82
Insula	I	36	15	12	7	−4.17
Anterior Cingulate cortex	C	−6	30	18	5	−3.69
Middle frontal gyrus	C	−27	36	24	8	−3.40
Superior medial gyrus	I	9	36	48	5	−3.24
Parahippocampal gyrus	I	27	0	−30	6	−3.08
Inferior temporal gyrus	C	−48	−48	−18	3	−3.00
Middle temporal gyrus	I	57	−18	−15	8	−3.00
Middle temporal gyrus	C	−51	−9	−15	12	−3.16
Amygdala	I	30	3	−15	12	−3.23
Middle temporal gyrus	I	57	−9	−12	6	−3.77
Insula	I	30	18	−9	6	−3.11
Insula	C	−27	24	3	8	−3.64
Middle frontal gyrus	I	39	45	0	6	−3.62
Middle frontal gyrus	I	33	33	24	6	−3.33
M1	I	48	−12	48	3	−3.04
Dorsal premotor cortex	C	−45	6	45	6	−3.15
Superior medial gyrus	C	0	30	57	6	−3.03
S1	I	42	−24	63	3	−3.04
Middle frontal gyrus	C	−36	9	63	12	−3.73
Fusiform gyrus	C	−33	−12	−27	4	−3.14
Middle temporal gyrus	I	51	−3	−21	8	−3.59
Middle temporal gyrus	I	57	−3	−21	8	−3.24
Hippocampus	C	−30	−21	−15	4	−3.20
Amygdala	C	−21	−6	−15	4	−3.12
Superior temporal gyrus	C	−51	12	−15	4	−3.24
Hippocampus	I	33	−18	−12	2	−3.83
Superior temporal gyrus	I	48	−9	−12	2	−4.00
Middle temporal gyrus	I	63	−33	−9	8	−3.00
Middle temporal gyrus	C	−51	−66	−3	4	−3.24
Superior temporal gyrus	I	66	−18	3	2	−3.13
Superior temporal gyrus	I	57	−6	3	4	−3.52
Inferior frontal gyrus	C	−57	12	6	2	−3.20
Insula	C	−36	−9	15	4	−3.03
Inferior frontal gyrus	I	45	3	21	8	−3.02
Inferior frontal gyrus	C	−39	15	21	8	−3.55
Anterior cingulate cortex	I	6	27	21	4	−3.24
Superior occipital gyrus	C	−18	−69	24	2	−3.13
Superior medial gyrus	C	−9	42	27	4	−3.08
Inferior frontal gyrus	C	−51	27	30	4	−3.00
Superior occipital gyrus	I	27	−66	33	4	−2.99
Superior occipital gyrus	I	−54	21	33	4	−3.15
Precuneus, middle cingulate cortex	C	−15	−51	39	8	−3.12
Dorsal premotor cortex	C	−54	−9	42	2	−2.98
Superior medial gyrus	C	−6	45	42	2	−3.72
Inferior parietal lobule	C	−57	−36	48	2	−3.08
Inferior parietal lobule	C	−42	−33	51	4	−4.47
Middle frontal gyrus	C	−21	27	51	8	−3.31
Superior medial gyrus	I	9	39	54	4	−3.23
Superior frontal gyrus	C	−21	24	57	4	−2.98
Superior frontal gyrus	I	15	33	57	8	−2.99
Superior frontal gyrus	C	−21	15	60	4	−2.99
Superior frontal gyrus	C	−30	−3	69	4	−3.18
Superior frontal gyrus	C	−15	15	69	8	−2.99
Cerebellum – crus II, VIIb, VIIIa, Dentate Nucleus	C	24	−68	−47	262	−3.08
Cerebellum – crus I, crus II	C	16	−78	−33	26	−3.17
Cerebellum – VIIIb, X	I	−14	−40	−51	10	−3.06
Cerebellum – crus I	C	42	−66	−29	6	−2.99
Cerebellum – dentate nucleus	I	−12	−68	−35	2	−2.99

Only brain regions surviving a height threshold of *p* < 0.005 (cluster size correction using 3dClustSim in AFNI providing an FWE-corrected *p* < 0.05). MNI coordinates for the voxel with the highest intensity in each cluster are provided. C, contralateral to side producing force; I, ipsilateral to side producing force; MNI, Montreal Neurological Institute.

Partial correlation analyses were run in PD between the force measures that differed between groups (i.e., rate of force increase, rate of force decrease) and the PSC in motor regions where group differences were found: contralateral globus pallidus external segment, contralateral M1 hand area and foot area, contralateral pre-SMA (cluster extending into the superior frontal gyrus), and the cluster in the ipsilateral cerebellum spanning lobules crus II, VIIb, VIIIa and the dentate nucleus. The partial correlations controlled by the MVC force indicated no relationship between the rate of force increase in PD and the PSC in any of the motor regions (*p* values > 0.05, Supplementary Table S1). However, a negative correlation was found between the rate of force decrease in PD and the PSC in the contralateral pre-SMA (cluster extending into the superior frontal gyrus) (*r* = −0.499, *p* = 0.030; Supplementary Table S1). That is, the greater the fMRI activity within the contralateral pre-SMA in PD, the faster the rate of relaxation of the hand and foot. There was no relationship between the rate of force decrease and the PSC in the other motor regions (i.e., contralateral globus pallidus external segment, M1 hand and foot areas, and ipsilateral cerebellum) (*p* values > 0.05; Supplementary Table S1).

## 4. Discussion

In this study, we conducted the first investigation of PD-related changes in fMRI activity during a multi-limb task, in which individuals generated force simultaneously using both their ipsilateral hand and foot. The study yielded several noteworthy findings. First, PD were able to perform simultaneous isometric contractions of the ipsilateral hand and foot at the designated rate (i.e., 15% MVC, where MVC represents the maximum voluntary contraction force produced by both limbs combined). However, it was observed that PD exhibited slower rates of contraction and relaxation for the two limbs compared to the control group. Second, the multi-limb coordination task revealed a mixed pattern of underactivation and overactivation, with reduced fMRI activity in nuclei of the basal ganglia and M1 in PD relative to controls, and increased fMRI activity in more rostral motor regions such as pre-SMA, as well as the lateral cerebellum. Finally, the greater the fMRI activity within pre-SMA in PD, the faster the rate of relaxation of the ipsilateral hand and foot.

### 4.1. PD-related changes in multi-limb force control

The results of the force analysis demonstrated that both groups coordinated the ipsilateral hand and foot in a similar manner, as indicated by the absence of significant group differences in the time lag between the onset of force in each limb. Notably, the observed lag between the hand and foot was minimal, on the order of several milliseconds, which suggests synchronization between the hand and foot movement. However, the variability of the time lag in PD appears to be greater than in HC, as shown in Table 2. Although this measure was not a focus of the analysis, it could indicate synchronization issues in some PD. Since the participants included in this study were primarily in the early stages of the disease, as determined by the Hoehn and Yahr staging system, it would be valuable for future studies to investigate whether synchronization deficits become more pronounced in the later stages of the disease. The analysis of force distribution showed that, overall, there were no significant group differences in the contributions of the hand and foot to the total force generated (*p*-values >0.05; Table 2). However, some slightly different within-group differences were observed. The current experimental design is not able to determine potential factors that may explain the distribution of forces generated by the hand and foot during this isometric coordination task. For instance, differences in muscle strength between limbs may influence force distribution. Stiffness which is common in PD can also impact the ability to modulate force distribution. Finally, it is important to emphasize that participants were instructed to aim for roughly equal force production with both the hand and the foot during the task. However, they were not provided specific instructions regarding the strategies they could employ to achieve this balance.

Importantly, the absence of significant group differences in force amplitude indicates that both PD and controls met the task’s force requirements effectively. The similarity in force production during the scanning session is desirable because the BOLD fMRI signal strength is known to scale with force output (Spraker et al., 2007; Noble et al., 2011). To prevent potential misinterpretation of group differences, we deliberately had participants produce submaximal forces at a preset % of their MVC (i.e., 15%), ensuring consistency in force demands. This approach aligns with established standards in force control fMRI studies, enabling accurate comparisons between PD and controls while isolating disease effects from muscle strength variations. Regarding the precision and variability of the multi-limb movements, no significant differences were observed between groups. This contrasts with previous findings from a brain imaging study examining homologous limbs, where individuals with PD exhibited greater errors and variability during bimanual movements compared to controls (Wu et al., 2010). However, these differences were specific to anti-phase movements and not observed during in-phase movements (Wu et al., 2010). In our study, participants performed an isometric multi-limb force control task that required applying force to a sensor using the thumb, while simultaneously dorsiflexing the ankle. Isotonic contractions of the ipsilateral hand and foot in the opposite direction in the sagittal plane, although not employed in this study due to the potential impact of varying movement amplitudes on the BOLD fMRI signal, might present a more challenging and comparable scenario to the anti-phase bimanual movements examined in the aforementioned study.

A noteworthy finding that emerged from the analysis of the force data pertains to the changes in PD during the multi-limb task in the rates of force increase and force decrease. These results are not surprising as it is commonly recognized that basal ganglia dysfunction and functional changes in other regions of the motor system disrupt fine-tuned control of muscle activity in PD, leading to slowed rates of force modulation (Stelmach and Worringham, 1988; Stelmach et al., 1989; Kunesch et al., 1995; Vaillancourt et al., 2004, 2006; Park and Stelmach, 2007; Prodoehl et al., 2009; Spraker et al., 2010; Neely et al., 2013b; Chung et al., 2023b). In the past, these changes were found to impact various motor tasks, such as grasping or adjusting force during rapid movements, and may contribute to the motor difficulties experienced by individuals with PD (Stelmach and Worringham, 1988; Stelmach et al., 1989; Kunesch et al., 1995; Park and Stelmach, 2007; Stegemöller et al., 2009, 2010; Neely et al., 2013b; Chung et al., 2018, 2023b). Our study provides further confirmation of previous findings from single-limb studies using this isometric force control paradigm, which demonstrated that individuals with PD exhibit slowness in force generation during isometric contractions and force release upon cessation of contraction (Neely et al., 2013b; Chung et al., 2023b). Although direct comparisons between the results of this study and unimanual investigations are precluded, it appears that the rates of force increase and decrease may be more pronounced when the task engages multiple limbs. In conclusion, the analysis of the force data provides compelling evidence that the impact of PD on movement speed extends beyond simple single-limb movements.

### 4.2. PD-related changes in brain activity

The multi-limb coordination task unveiled a complex pattern of neural activity as assessed through fMRI. Notably, we found diminished motor-related activity within the basal ganglia and M1 hand and foot areas, and an increase in motor-related activity in motor regions located in the prefrontal cortex and cerebellar hemisphere, which are recognized for their involvement in motor planning and coordination.

First, it is essential to highlight the existence of several fMRI studies in PD that have utilized tasks involving rapid contraction and relaxation to generate 15% of MVC. Concurrently, it is important to note that investigations into force control deficits and the corresponding changes in brain activity have been conducted independently for the hand and foot (Prodoehl et al., 2010; Spraker et al., 2010; Burciu et al., 2015, 2016a,b; Neely et al., 2015; Planetta et al., 2015; Chung et al., 2018, 2023a). Thus far, previous studies have primarily focused on analyzing isolated movements of the hand and foot, while coordinated movements involving both the hand and foot have not yet been examined. Regarding the findings, multiple upper limb studies have demonstrated reduced fMRI activity in multiple nuclei of the basal ganglia and M1 across in PD compared to controls (Prodoehl et al., 2010; Spraker et al., 2010; Burciu et al., 2015, 2016a,b; Neely et al., 2015; Planetta et al., 2015). Furthermore, this pattern of underactivation has been recently demonstrated not only during hand movements but also during foot movements. Specifically, in one of our recent studies investigating the activation of brain regions during ankle dorsiflexion in PD, we found that the basal ganglia nuclei and M1 (foot area) are hypoactive in PD compared to controls (Chung et al., 2023a). The precise effects of PD on complex movements that involve coordinated actions of the hand and foot, as well as the activity of the basal ganglia and cortical motor areas, remain largely unknown. Through this study, which is the first to investigate the neural correlates of multi-limb force control deficits in PD, we contribute to the existing literature by demonstrating that the basal ganglia and motor cortex exhibit underactivity not only during single-limb movements (i.e., isolated movements of the hand or the foot) but also during multi-limb movements. Specifically, the globus pallidus external segment along with the hand and foot areas in M1 are underactive in PD during simultaneous contractions and relaxations of the more affected (ipsilateral) hand and foot. The dysfunctional activity of the hand and foot areas in M1 was expected and is believed to be a consequence of the disrupted communication between the basal ganglia and M1 (G E Alexander et al., 1986, Albin et al., 1989, Obeso and Lanciego, 2011). In PD, the degeneration of dopaminergic neurons in the substantia nigra pars compacta leads to an imbalance in the direct and indirect pathways within the basal ganglia-cortical loop (G E Alexander et al., 1986, Obeso and Lanciego, 2011). This disrupts the signals reaching the hand and foot areas in M1 (Obeso and Lanciego, 2011), leading to altered neural activity which in the context of the multi-limb task used here translates into impaired force control during simultaneous movements of the upper and lower limbs.

Our study also revealed a novel and important association in PD between the fMRI activation levels in a cluster encompassing the contralateral pre-SMA and portions of the contralateral superior frontal gyrus and speed-related measures. Specifically, PD with higher fMRI activity in this premotor/prefrontal area exhibited a faster rate of force decrease during the multi-limb coordination task. The pre-SMA, situated at the interface of the prefrontal and motor systems, is known to be involved in various brain functions including motor planning and coordination (Matsuzaka et al., 1992; Nachev et al., 2007). Previous research has highlighted its role in integrating the temporal order of multiple movements in a motor sequence and inhibitory control of actions (Nakajima et al., 2022). The multi-limb task employed here is expected to be relatively simple and automatic, requiring minimal attentional resources in healthy older adults. However, individuals with PD may experience difficulties in executing such tasks automatically. This difficulty could be attributed to dopaminergic dysfunction. Previously, an fMRI study investigating the neural mechanisms of movement automaticity in PD found that patients had greater activity in multiple brain regions including the premotor cortex, cerebellum, prefrontal and parietal cortices compared with control participants when performing automatic movements of the upper limb (Wu and Hallett, 2005). Together, these findings suggest that additional attentional and neural resources may be necessary for optimal task performance (here, coordinating both hand and foot to produce 15% MVC). The observed increased activation of the pre-SMA in this study underscores its potential as a compensatory neural resource in PD. If we assume that the pre-SMA functions as a compensatory mechanism (further investigations are necessary to confirm this hypothesis), it will be important to determine if individuals found in the more advanced stages of PD will encounter a limitation in the available neural resources that can be recruited to support task performance.

Finally, the simultaneous multi-limb force control task of the ipsilateral hand and foot also revealed increased fMRI activity in PD compared to HC within the ipsilateral cerebellum. Specifically, regions of increased activity in the ipsilateral cerebellum in PD included lobules crus I-II, VIIb, VIIIa, and the dentate nucleus. Similar to the pre-SMA, the lateral portions of the cerebellum are believed to be involved in the more cognitive aspects of motor behavior, involving the integration of sensory information and precise temporal coordination of movements (Manto et al., 2012). Importantly, the findings of the current study deviate from previous fMRI research that reported reduced cerebellar activity in individuals with PD during unimanual instrumented tasks (Burciu et al., 2015, 2016a; Planetta et al., 2015; Herz et al., 2021). Similarly, in a recent fMRI experiment where PD performed isometric ankle dorsiflexion at 15% of MVC, it was observed that the cerebellum is less active in PD compared to control participants (Chung et al., 2023a). Considering the approximately 75% overlap in participants between the ankle dorsiflexion study (Chung et al., 2023a) and the current multi-limb study, along with the variations in task complexity, it is likely that task demands have a distinct impact on cerebellar activation in PD. It is possible that both the cerebellum and regions such as pre-SMA demonstrate an overactivation when task demands increase, such as in tasks that require the coordination of multiple limbs compared to tasks that involve only single-limb movements (e.g., contracting/relaxing the hand and foot together vs. the hand and foot separately). Considering that the cerebellum primarily utilizes GABA as its main neurotransmitter (Takayama, 2005), it could be that the observed patterns of under- and overactivation, along with the corresponding motor symptoms, may be attributed to a complex yet altered interplay between dopaminergic and non-dopaminergic systems. Currently, there is a growing body of evidence suggesting that PD is a multisystem neurodegenerative disorder (Błaszczyk, 2016). Finally, it will be important that future studies investigate more complex coordinated movements that are more reflective of everyday activities in PD (e.g., anti-phase and multi-joint movements), and movements that involve the generation of greater forces.

In summary, this study provides insights into the multi-limb force control deficits and associated functional activation maps in individuals with PD compared to HC. Despite diminished motor-related activity within the basal ganglia-cortical loop, PD participants exhibited a distinct pattern of widespread BOLD fMRI signal overactivation in motor planning and coordination regions of the prefrontal cortex and lateral cerebellum. These findings highlight possible compensatory mechanisms that may contribute to task performance in PD. Investigating the cortico-basal ganglia and cortico-cerebellar pathways during multi-limb movements not only sheds more light on how these pathways operate in a more complex motor context but can also contribute to the optimization of exercise programs which are an essential component of PD management. Tailoring exercise regimens to target specific brain regions involved in multi-limb control may lead to more effective therapies for improving overall motor function and coordination in PD patients.

## Data availability statement

The raw data supporting the conclusions of this article will be made available by the authors, without undue reservation.

## Ethics statement

The studies involving humans were approved by University of Delaware Institutional Review Board. The studies were conducted in accordance with the local legislation and institutional requirements. The participants provided their written informed consent to participate in this study.

## Author contributions

JC: investigation, formal analysis, writing – original draft, and review and editing. AB and IM: investigation and writing – review and editing. JM, CK, and JJ: writing – review and editing. RB: funding acquisition, conceptualization, methodology, investigation, formal analysis, writing – original draft, and review & editing. All authors contributed to the article and approved the submitted version.

## Footnotes

^1^ https://afni.nimh.nih.gov

^2^ https://www.fil.ion.ucl.ac.uk/spm/

^3^ https://www.diedrichsenlab.org/imaging/suit.htm

## Supplementary material

Supplementary Table S1 The table describes the results of the partial correlation between the force measures and the PSC (controlling for MVC) in motor regions where group differences were found: contralateral GPe, contralateral M1 hand/foot area, contralateral pre-SMA (cluster extending into the superior frontal gyrus), and the cluster in the ipsilateral cerebellum spanning lobules Crus II, VIIb, VIIIa, and the dentate nucleus. Abbreviations: C = contralateral to side producing force; GPe = globus pallidus external segment; I = ipsilateral to side producing force; M1 = primary motor cortex; PSC = percent signal change; SMA = supplementary motor area.
