# Supplementary material for: fMRI changes during multi-limb movements in Parkinson’s disease
Source: Front Hum Neurosci. 2023 Nov 9;17:1248636. doi: 10.3389/fnhum.2023.1248636 (PMC10665733; doi:10.3389/fnhum.2023.1248636)
Supplement: Supplementary Table S1 — The table describes the results of the partial correlation between the force measures and the PSC (controlling for MVC) in motor regions where group differences were found: contralateral GPe, contralateral M1 hand/foot area, contralateral pre-SMA (cluster extending into the superior frontal gyrus), and the cluster in the ipsilateral cerebellum spanning lobules Crus II, VIIb, VIIIa, and the dentate nucleus. Abbreviations: C = contralateral to side producing force; GPe = globus pallidus external segment; I = ipsilateral to side producing force; M1 = primary motor cortex; PSC = percent signal change; SMA = supplementary motor area. [file Table_1.docx]

**Supplementary Table 1**

| **Force**  **Variable** | **Partial Correlation Parameters** | **C Pre-SMA/SFG** | **C M1 Hand** | **C M1 Foot** | **C GPe** | **I Cerebellum**  **(VIIb, Crus II, VIIIa, Dentate Nucleus)** |
| --- | --- | --- | --- | --- | --- | --- |
| Rate of Force Increase | r | -0.098 | 0.092 | 0.072 | -0.105 | -0.108 |
|  | *p*-value | 0.690 | 0.707 | 0.769 | 0.668 | 0.661 |
| Rate of Force Decrease | r | -0.499 | -0.080 | 0.469 | 0.357 | 0.431 |
|  | *p*-value | 0.030 | 0.746 | 0.106 | 0.134 | 0.066 |
